# Supplementary material for: Structural and dynamic insights into the biased signaling mechanism of the human kappa opioid receptor
Source: Nat Commun. 2025 Oct 28;16:9392. doi: 10.1038/s41467-025-64882-1 (PMC12569041; doi:10.1038/s41467-025-64882-1)
Supplement: Supplementary file 1 — Supplementary Information [file 41467_2025_64882_MOESM1_ESM.pdf]

# Supplementary Information

**Structural and Dynamic Insights into the Biased Signaling Mechanism of the Human Kappa Opioid Receptor**

Chiyo Suno-Ikeda[1,†], Ryo Nishikawa[2,†], Riko Suzuki[3,†], Shun Yokoi[4,5], Seiya Iwata[2], Tomoyo Takai[1], Takaya Ogura[6], Mika Hirose[7], Akihisa Tokuda[5], Risako Katamoto[5], Akitoshi Inoue[1], Eri Asai[1], Ryoji Kise[3], Yukihiko Sugita[8,9,10], Takayuki Kato[7], Hiroshi Nagase[5], Ayori Mitsutake[4], Tsuyoshi Saitoh[5,11], Kota Katayama[2,12], Asuka Inoue[3,6], Hideki Kandori[2,12], Takuya Kobayashi[1,13,*], Ryoji Suno[1,*]

[1] Department of Medical Chemistry, Kansai Medical University, Hirakata 573-1010, Japan.

[2] Department of Life Science and Applied Chemistry, Nagoya Institute of Technology, Showa-ku, Nagoya 466-8555, Japan

[3] Graduate School of Pharmaceutical Sciences, Kyoto University,606-8501, Japan

[4] Department of Physics, School of Science and Technology, Meiji University, 1-1-1 Higashi-Mita, Tama-ku, Kawasaki, Kanagawa 214−8571, Japan

[5] International Institute for Integrative Sleep Medicine (IIIS), University of Tsukuba, 1-1-1 Tennodai, Tsukuba, Ibaraki 305-8575, Japan.

[6] Graduate School of Pharmaceutical Sciences, Tohoku University, 6-3, Aoba, Aramaki, Aoba-Ku, Sendai, Miyagi 980-8578, Japan

[7] Institute for Protein Research, Osaka University, Suita 565-0871, Japan.

[8] Hakubi Center for Advanced Research, Kyoto University, Kyoto 606-8501, Japan.

[9] Institute for Life and Medical Sciences, Kyoto University, Kyoto 606-8507, Japan.

[10] Graduate School of Biostudies, Kyoto University, Kyoto 606-8507, Japan

[11] Faculty of Medicine, University of Tsukuba, 1-1-1 Tennodai, Tsukuba, Ibaraki 305-8575, Japan

[12] OptoBioTechnology Research Center, Nagoya Institute of Technology, Showa-ku, Nagoya 466-8555, Japan

[13] Japan Agency for Medical Research and Development (AMED), Core Research for Evolutional Science and Technology (CREST), 1-7-1 Otemachi, Chiyoda-ku, Tokyo 100-0004, Japan.

[†] **These authors contributed equally**

[*] **Corresponding Authors:**

Ryoji Suno

Department of Medical Chemistry, Kansai Medical University, Hirakata, 573-1010, Japan.

Email: suno.ryo@kmu.ac.jp

Takuya Kobayashi

Department of Medical Chemistry, Kansai Medical University, Hirakata, 573-1010, Japan.

Email: kobayatk@hirakata.kmu.ac.jp

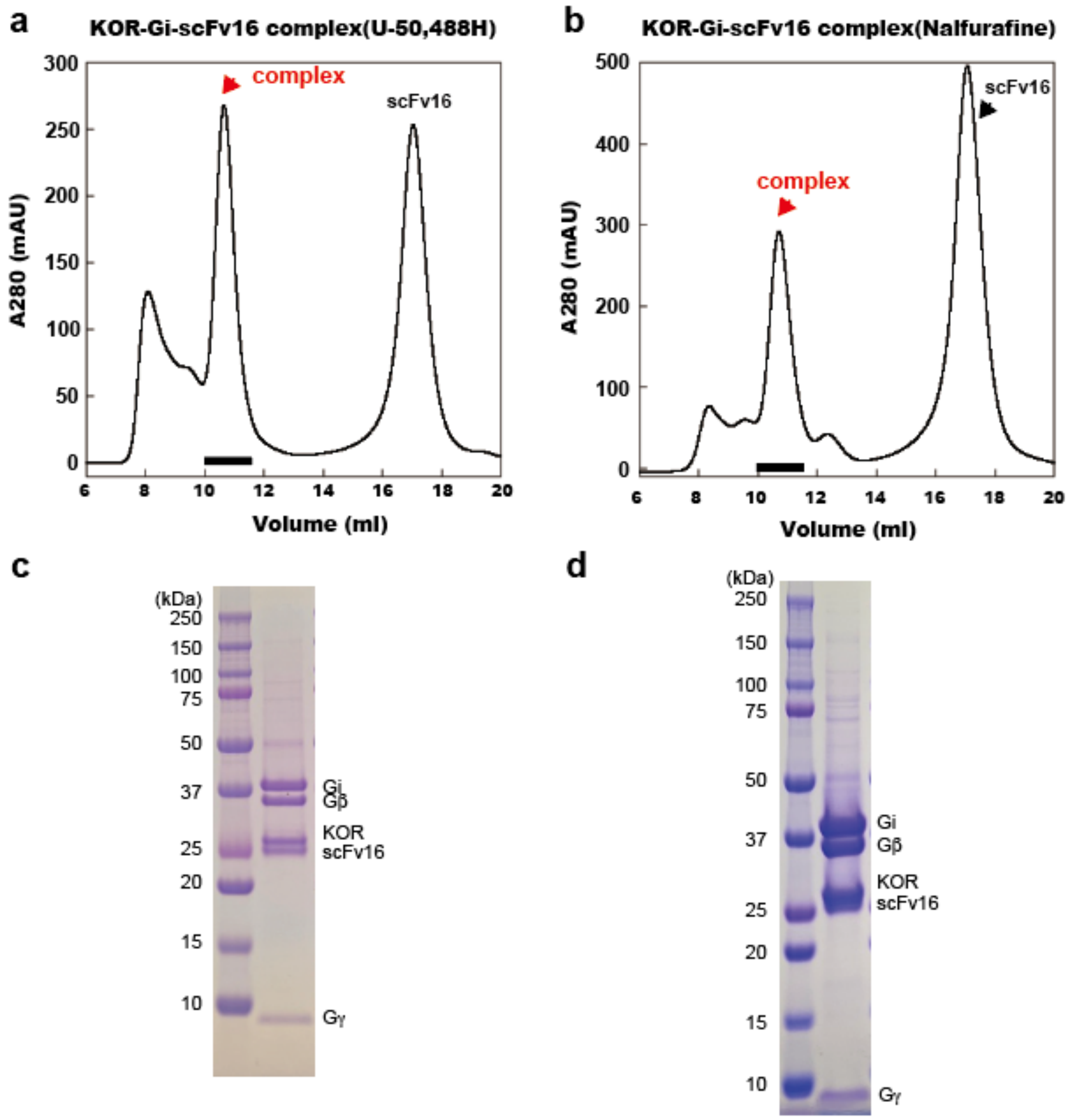


**Supplementary Figure 1.** Preparation of KOR-$G_i$ signaling complexes in U-50,488H-(**a,c**) and nalfurafine (**b,d**)-bound states. Chromatogram of gel filtration chromatography purification (**a, b**). SDS-PAGE of complex peaks in gel filtration chromatography (**c, d**).

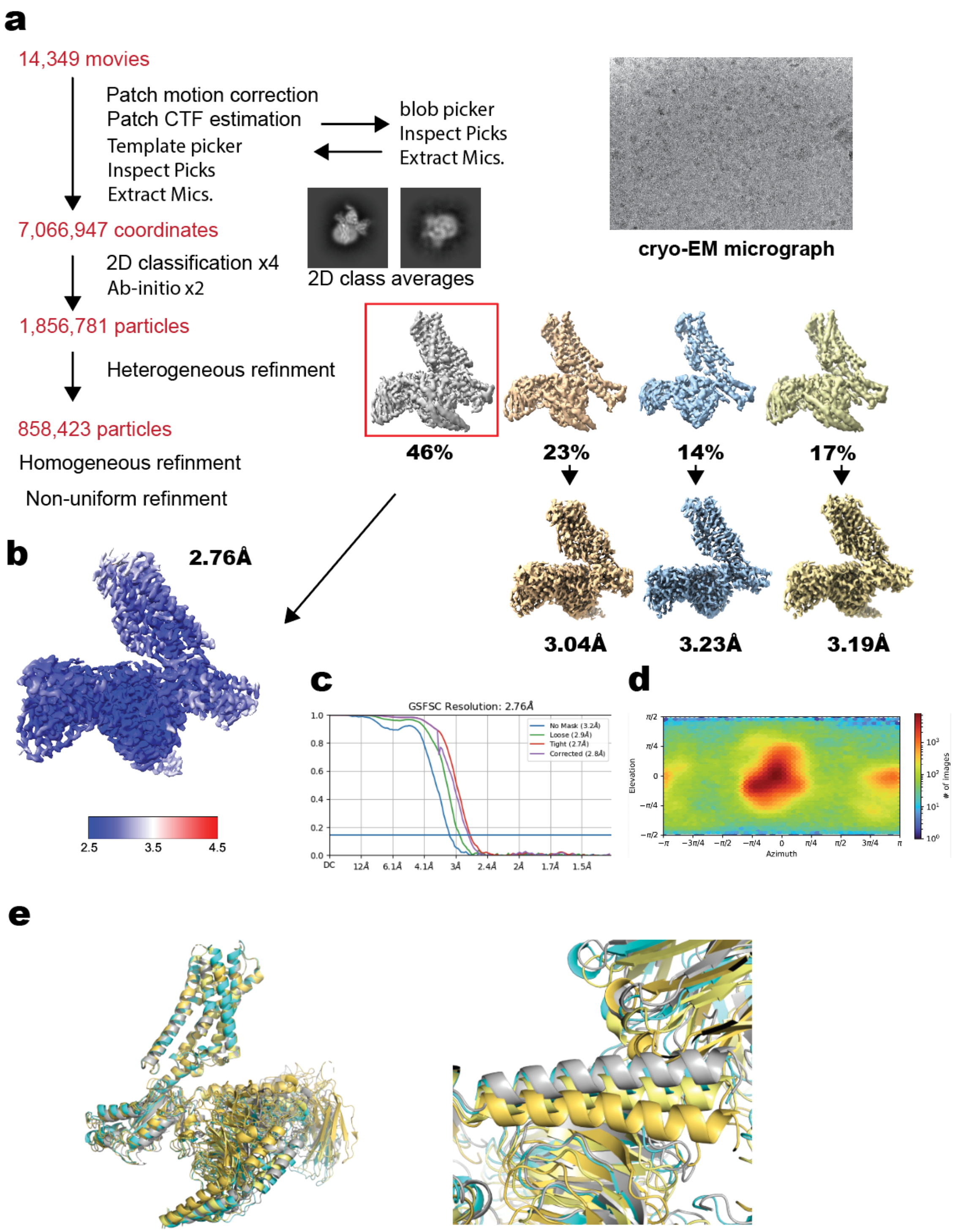


**Supplementary Figure 2. Cryo-EM data processing of nalfurafine-bound KOR-G$_i$ complex**

**a.** Workflow of the cryo-EM data processing. **b.** Local resolution maps for the non-uniform refinement of nalfurafine-bound KOR-G$_i$ signaling complex. (**c, d**) Gold standard FSC plots and directional distribution from the non-uniform refinement of the

nalfurafine-bound KOR-$G_i$ signaling complex calculated in cryoSPARC. **e.** Superimposed view of the KOR-$G_i$ signaling complex in four different nalfurafine binding states. Relative differences in G protein position (left) and N-terminal orientation (right) of G proteins due to superposition of receptor regions. The four KOR-$G_i$ signaling complex structures are at 2.76, 3.04, 3.23, and 3.19 Å resolution, respectively, and are shown in gray, orange, cyan, and yellow, respectively.

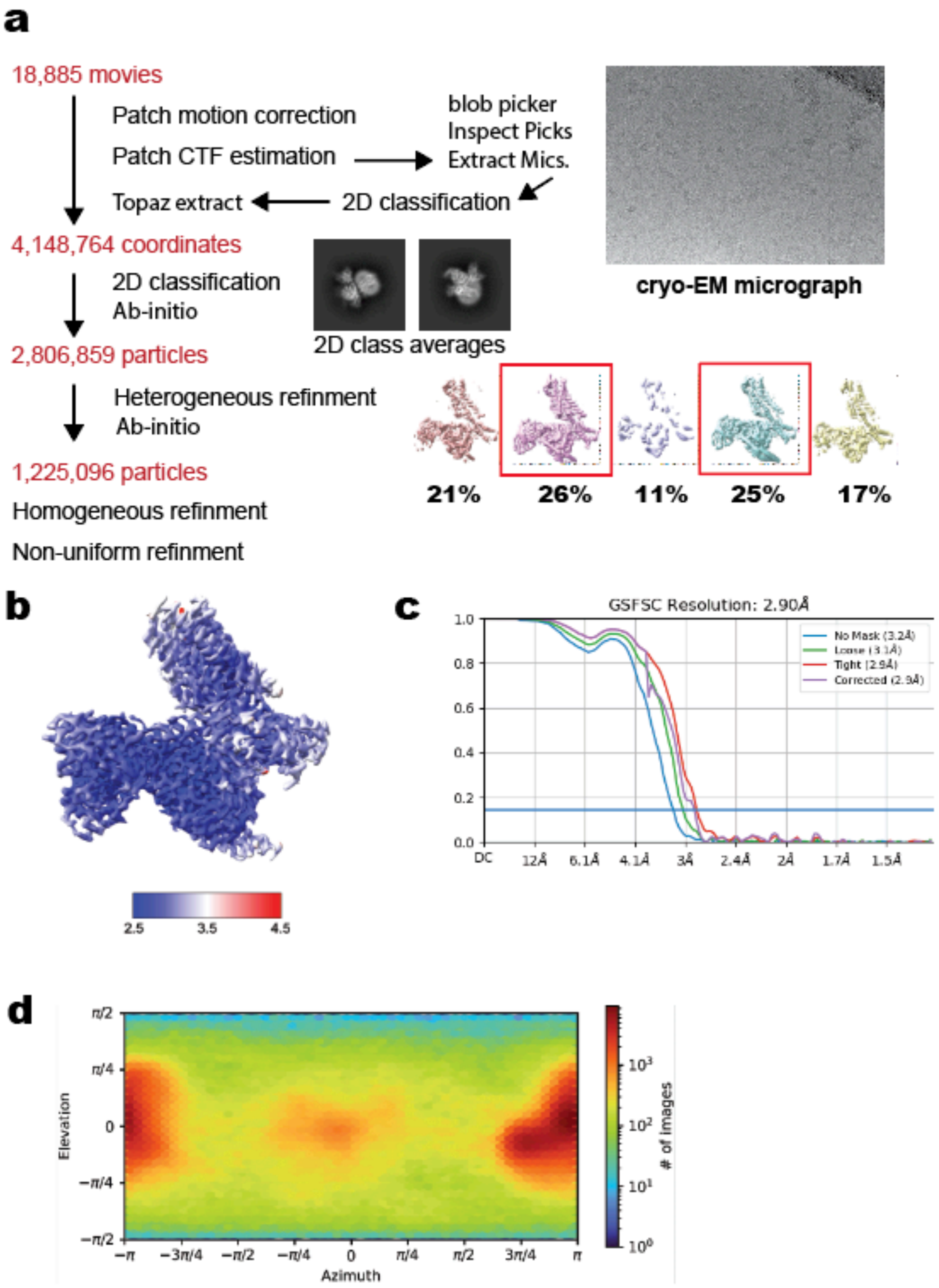


**Supplementary Figure 3. Cryo-EM data processing of U-50,488H-bound KOR-G$_i$ complex**

**a.** Workflow of the cryo-EM data processing. *Ab initio* reconstruction was performed by

mixing the particles in the two 3D classes indicated by red squares from the heterogeneous refinement results. **b.** Local resolution maps for the non-uniform refinement of U-50,488H-bound KOR-$G_i$ signaling complex. (**c, d**) Gold standard FSC plots and directional distribution from the non-uniform refinement of the U-50,488H-bound KOR-$G_i$ signaling complex calculated in cryoSPARC.

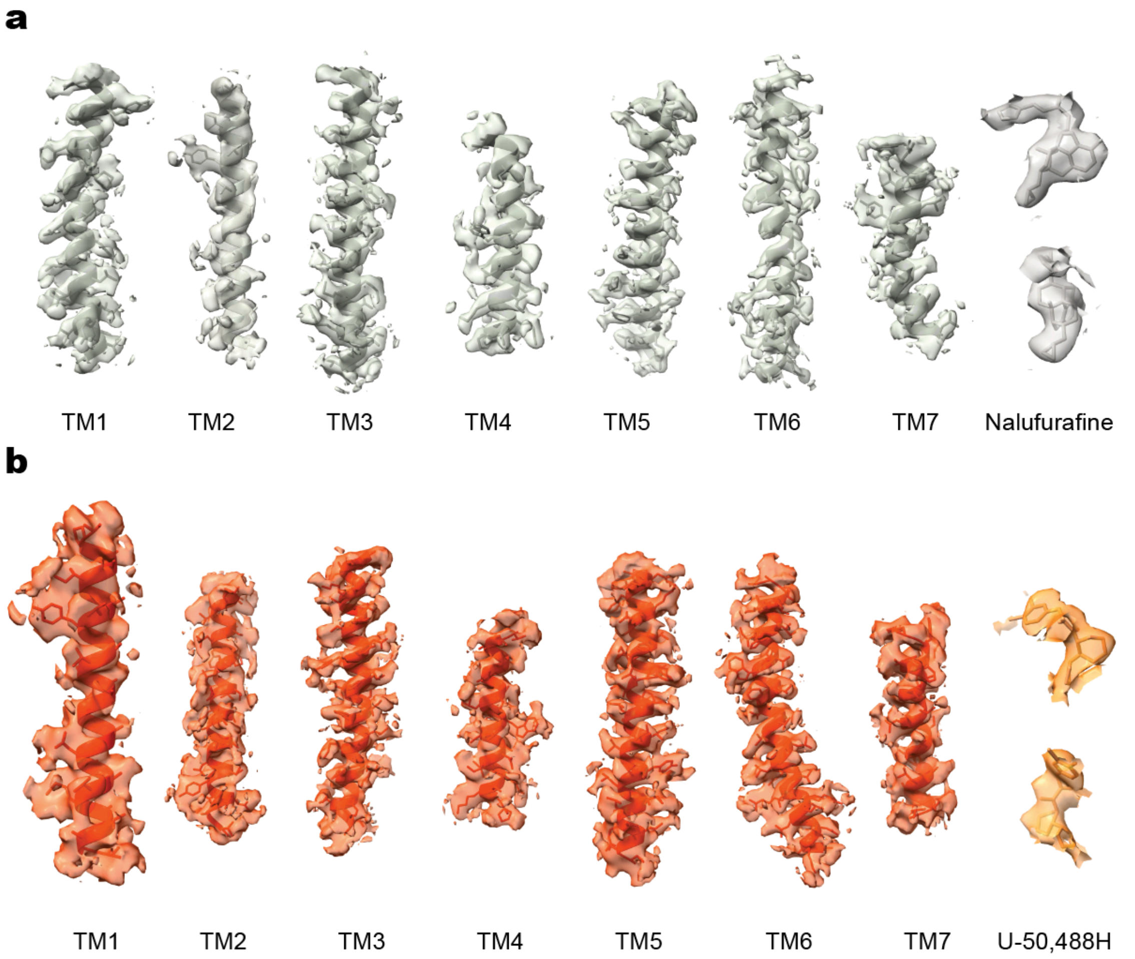


**Supplementary Figure 4. Cryo-EM density maps and models of the seven transmembrane helices (TM1-7) of nalfurafine- (a) or U-50,488H- (b) bound KOR.** Maps are shown in gray and orange, respectively.

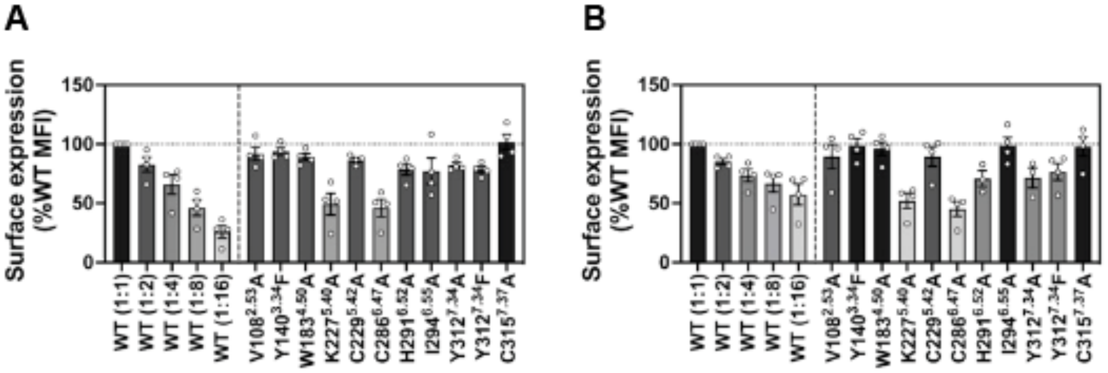


**Supplementary Figure 5. Surface expression of the KOR mutants analyzed by flow cytometry.**

(A) Surface expression levels of the KOR constructs used for G-protein dissociation assay. (B) Surface expression levels of the KOR constructs used for β-arrestin recruitment assay, which were fused with SmBiT in their C terminus. Data are presented as mean values ± SEM of 3–4 independent experiments with each performed in duplicate. The dilutions in the WT indicate volumes of the transfected WT KOR plasmid. Mutants with lowered surface expression levels are shown as grey bars whose color matches an equivalent expression level of plasmid-titrated WT.

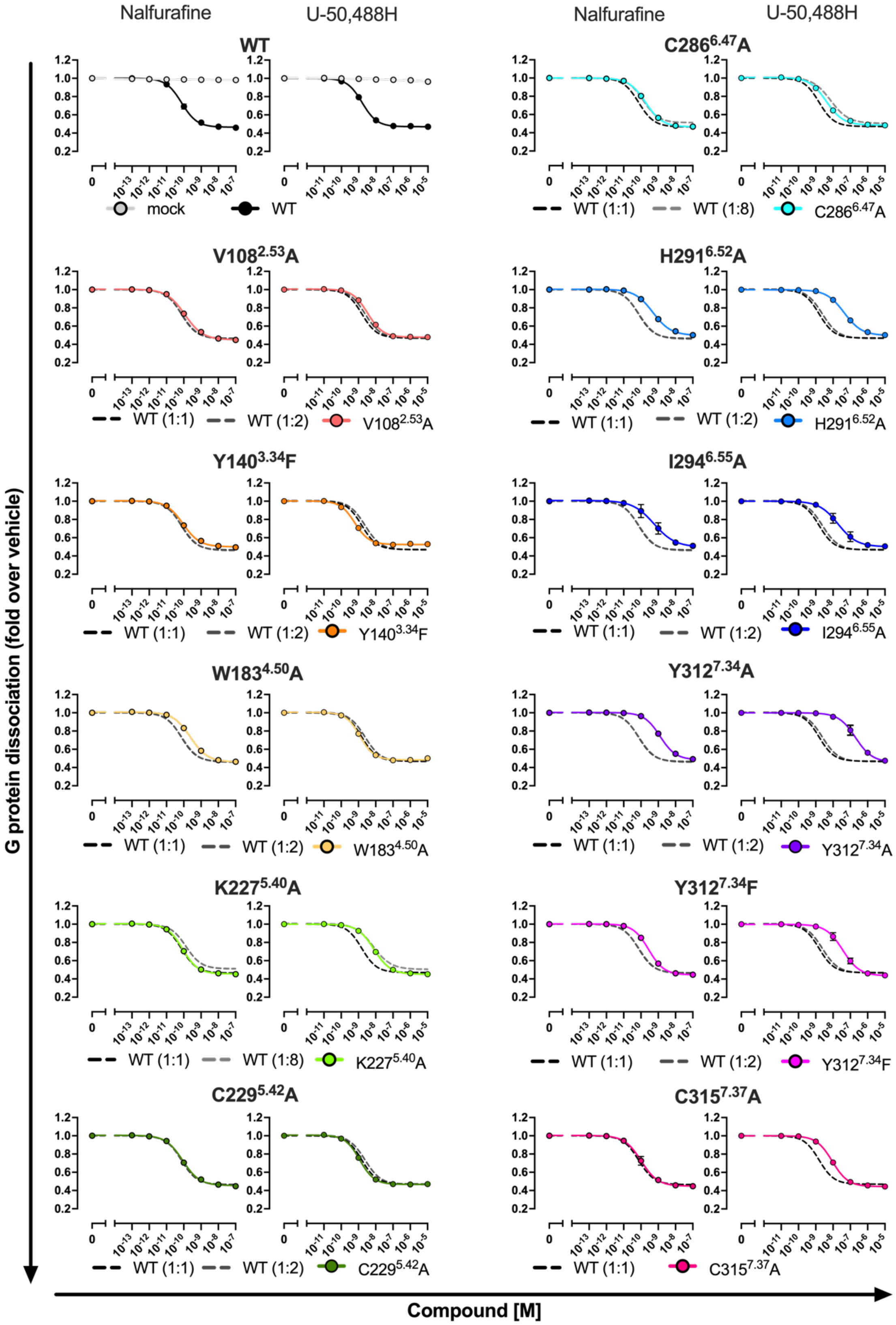


**Supplementary Figure 6. Concentration-response curves of the NanoBiT-G protein dissociation assay.**

Dashed lines in the mutant panels represent the wild-type (WT) KOR (1:1, 1:2, 1:4, 1:8, or 1:16) response. Data are presented as mean values ± SEM of three independent experiments with each performed in duplicate. Note that in many data points, the error bars are smaller than the size of the symbols. For mutans with reduced surface expression levels, expression-matched WT plasmid conditions are shown for comparisons (also see Supplementary Figure 5).

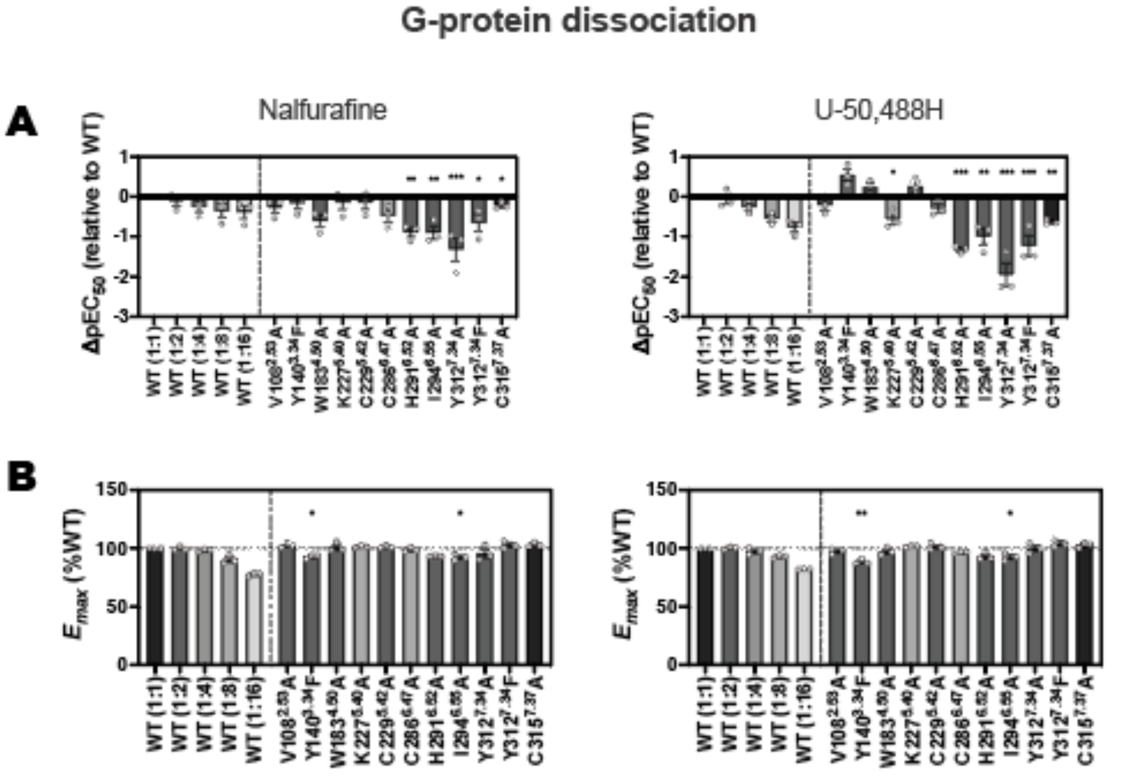


**Supplementary Figure 7. Pharmacological parameters for the $G_i$-coupling activity analyzed by the NanoBiT-G-protein dissociation assay.**

(A–B) For the individual experiments performed in parallel, data were normalized to WT KOR (1:1) and presented as $\Delta pEC_{50}$ (A) and $E_{max}$ (B). Data are presented as mean values ± SEM of 3–4 independent experiments with each performed in duplicate. The colors in the mutant bars correspond to the expression-matched WT conditions (see Supplementary Figure 5). Statistical analyses were performed using *t*-tests and the ordinary one-way ANOVA followed by the *Dunnett* test with the expression-matched (colored) WT response. ns, $p > 0.05$; $*p < 0.05$; $**p < 0.01$; $***p < 0.001$.

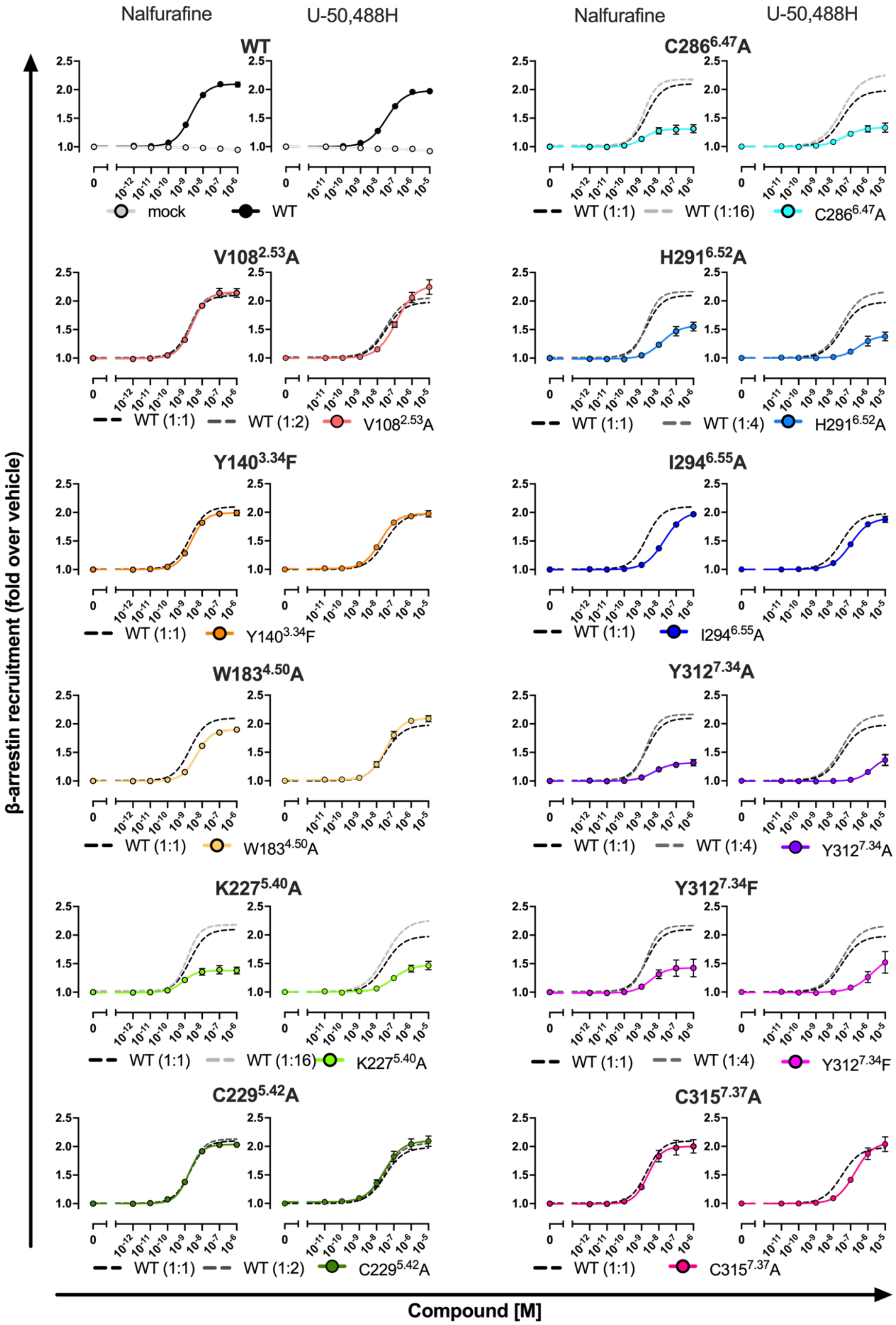


**Supplementary Figure 8. Concentration-response curves of the NanoBiT-β-arrestin recruitment assay.**

Dashed lines in the mutant panels represent the WT KOR (1:1, 1:2, 1:4, 1:8, or 1:16) response. Data are presented as mean values ± SEM of three independent experiments with each performed in duplicate. Note that in many data points, the error bars are smaller than the size of the symbols. For mutans with reduced surface expression levels, expression-matched WT plasmid conditions are shown for comparisons (also see Supplementary Figure 5).

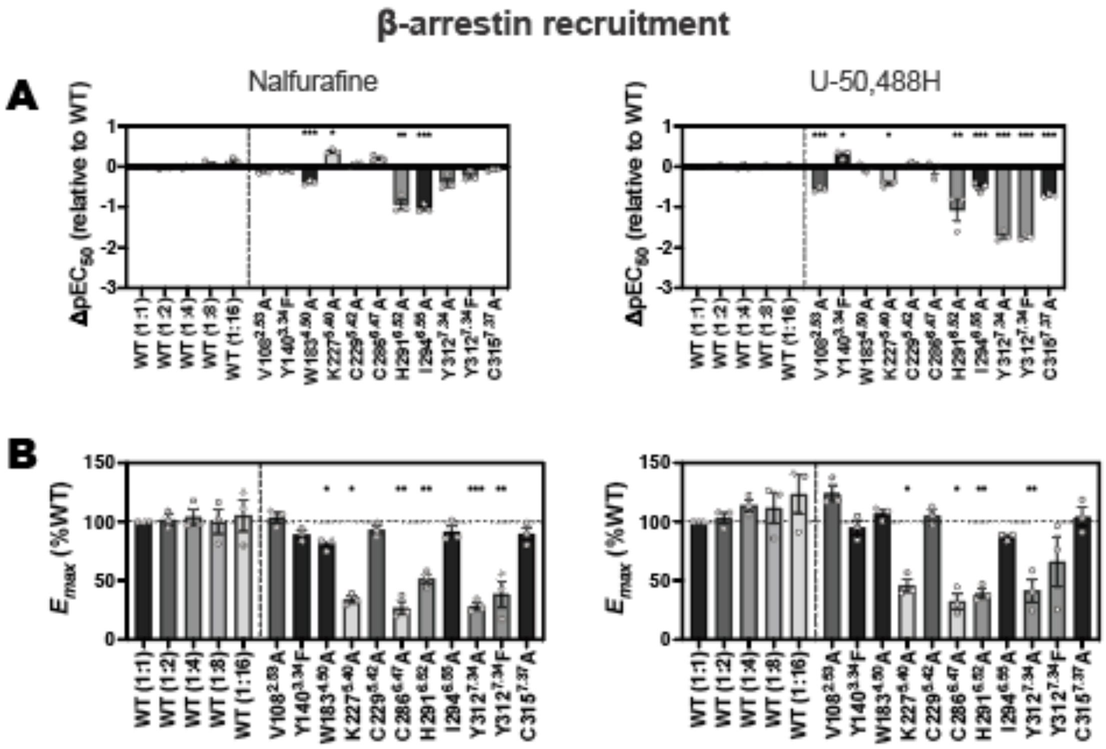


**Supplementary Figure 9. Pharmacological parameters for the β-arrestin2-recruiting activity analyzed by the NanoBiT-β-arrestin recruitment assay.**

**(A–B)** For the individual experiments performed in parallel, data were normalized to WT KOR (1:1) and presented as $\Delta pEC_{50}$ **(A)** and $E_{max}$ **(B)**. Data are presented as mean values ± SEM of 3–4 independent experiments with each performed in duplicate. The colors in the mutant bars correspond to the expression-matched WT conditions. Statistical analyses were performed using the ordinary one-way ANOVA followed by the *Dunnett* tests with the expression-matched (colored) WT response. ns, $p > 0.05$; *$p < 0.05$; **$p < 0.01$; ***$p < 0.001$.

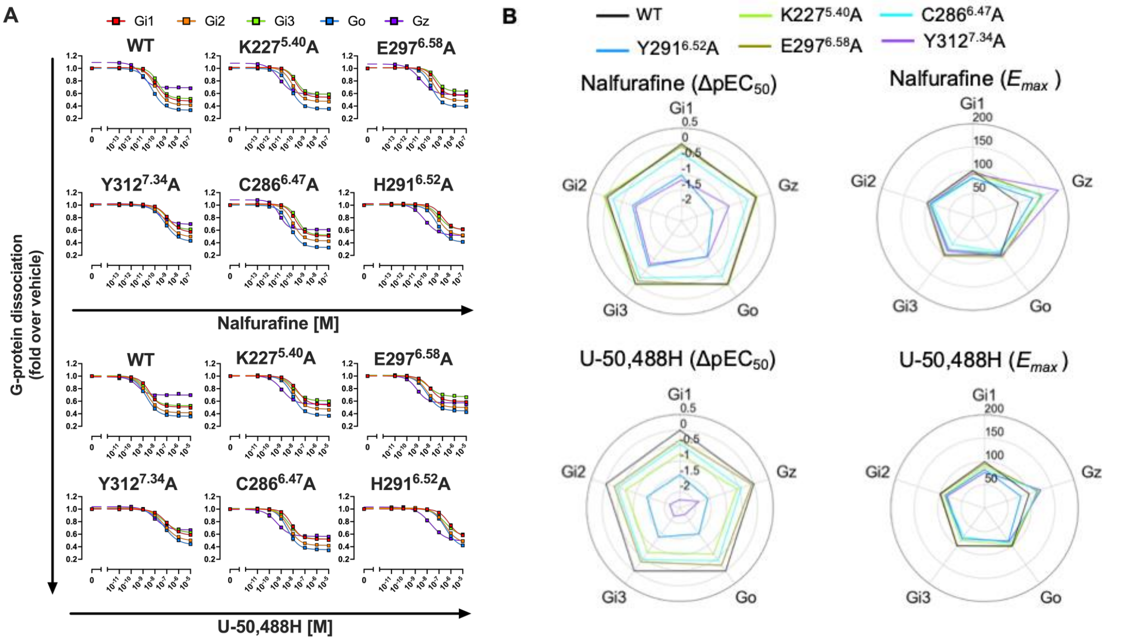


**Supplementary Figure 10. G-protein subtype selective effects of agonists and mutations**

**(A)** G-protein-mediated responses were examined with the NanoBiT-G-protein dissociation. (*n* = 1–2 independent experiment)

**(B)** Radar charts showing the pharmacological parameters of G-protein-subtype selectivity. The $pEC_{50}$ and $E_{max}$ values relative to those of WT KOR (1:1) were shown for each mutant.

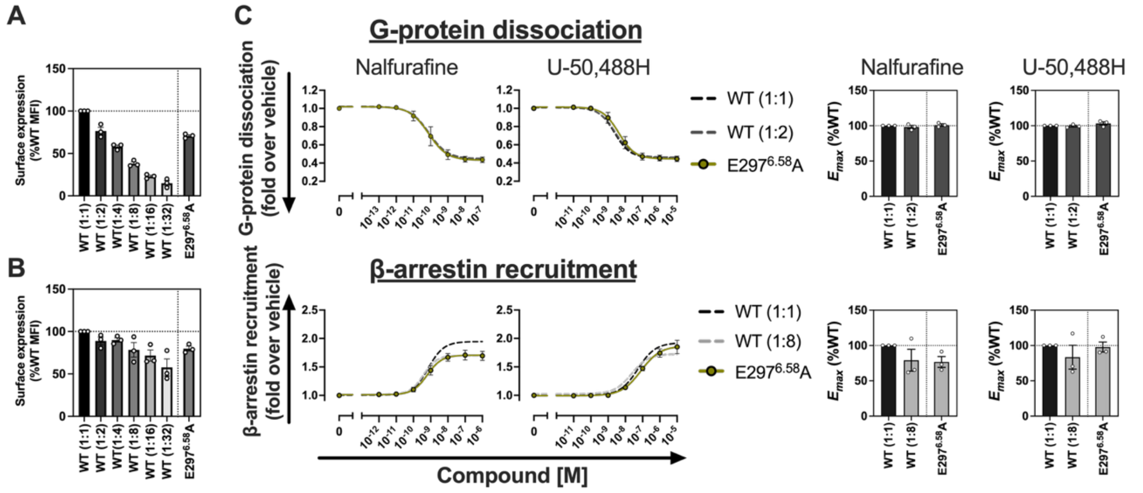


**Supplementary Figure 11. Evaluating the contribution of E297$^{6.58}$ in KOR signal transduction**

**(A–B)** Surface expression of KOR mutants was analyzed by flow cytometry. Data are presented as mean values ± SEM ($n$ = 3; dots). The results for KOR constructs used in G-protein dissociation **(A)** and β-arrestin recruitment **(B)** are shown. WT KOR plasmid amounts were serially diluted to match expression conditions. The bar graph for the E297$^{6.58}$A mutant is expression-matched (colored) to WT KOR. **(C)** G-protein- and β-arrestin-mediated responses were assessed using the NanoBiT-G-protein dissociation assay and NanoBiT-β-arrestin recruitment assay, respectively. Concentration-response curves for nalfurafine- and U-50,488H-induced responses are shown.

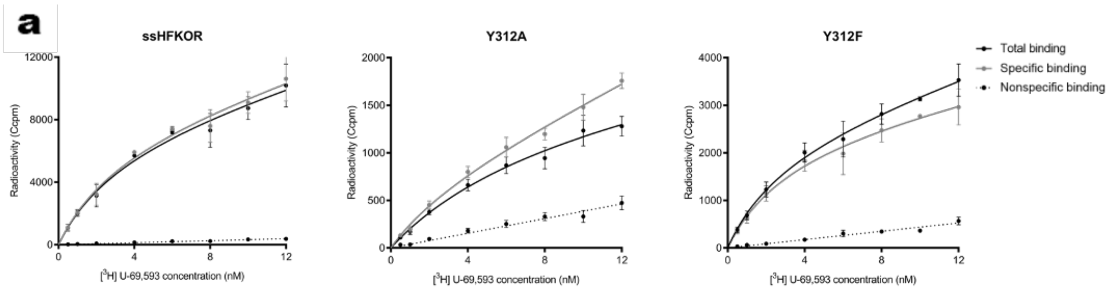


| | ssHFKOR (100 µg/ml) | Y312A (100 µg/ml) | Y312F (100 µg/ml) |
|---|---|---|---|
| $K_D$ value (nM) (95 % CI) | 7.15 (4.02 to 14.4) | 12.2 (8.04 to 20.2) | 6.23 (3.90 to 10.66) |

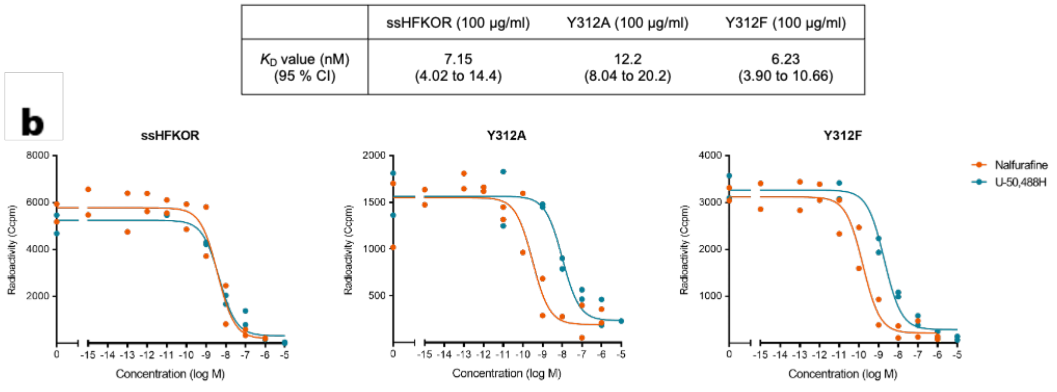


| | $K_i$ value (nM) | | |
|---|---|---|---|
| | ssHFKOR | Y312A | Y312F |
| Nalfurafine | 1.92 | 0.154 | 0.0795 |
| U-50,488H | 2.33 | 5.08 | 0.974 |

**Supplementary Figure 12. Ligand binding assay of KOR agonists against Y312$^{7.34}$A/F mutants. (a)** Saturation binding curves of the agonist [³H] U-69,593 for the wild type (WT), Y312$^{7.34}$A, and Y312$^{7.34}$F KOR variants. Total, nonspecific, and specific binding curves were obtained by non-linear regression analysis. Nonspecific binding was determined in the presence of 10 µM U-69,593. Specific binding was determined as the difference between total binding and nonspecific binding. Data are represented as the mean ± SEM. (error bars). The table below shows $K_d$ value, and 95% CI obtained from dose-response curves of three independent experiments (N = 3), each performed in triplicate. **(b)** Competitive binding curves of KOR agonists for the WT, Y312$^{7.34}$A, and Y312$^{7.34}$F KOR variants. Binding affinities of KOR agonists were measured by displacement of [³H] U-69,593. Data are presented as mean values ± SEM (error bars). The table below shows the $K_i$ value obtained from dose-response curves of two independent experiments (N = 2; shown as raw values, not included in statistical comparisons.).

–

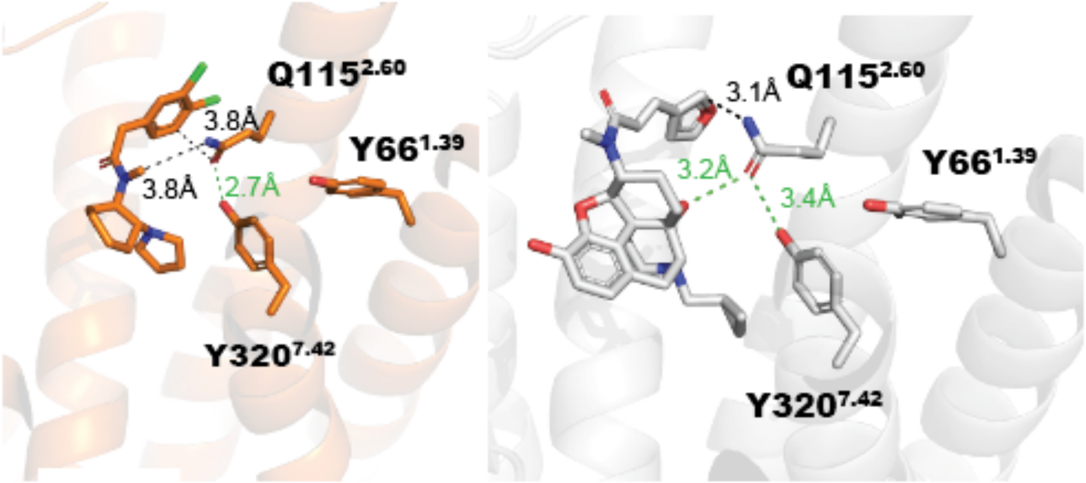


**Supplementary Figure 13. Binding modes of residue Q115$^{2.60}$ with ligands in the KOR–Gi signaling complexes.**

The U-50,488H–bound complex is shown in orange and the nalfurafine–bound complex is shown in gray.

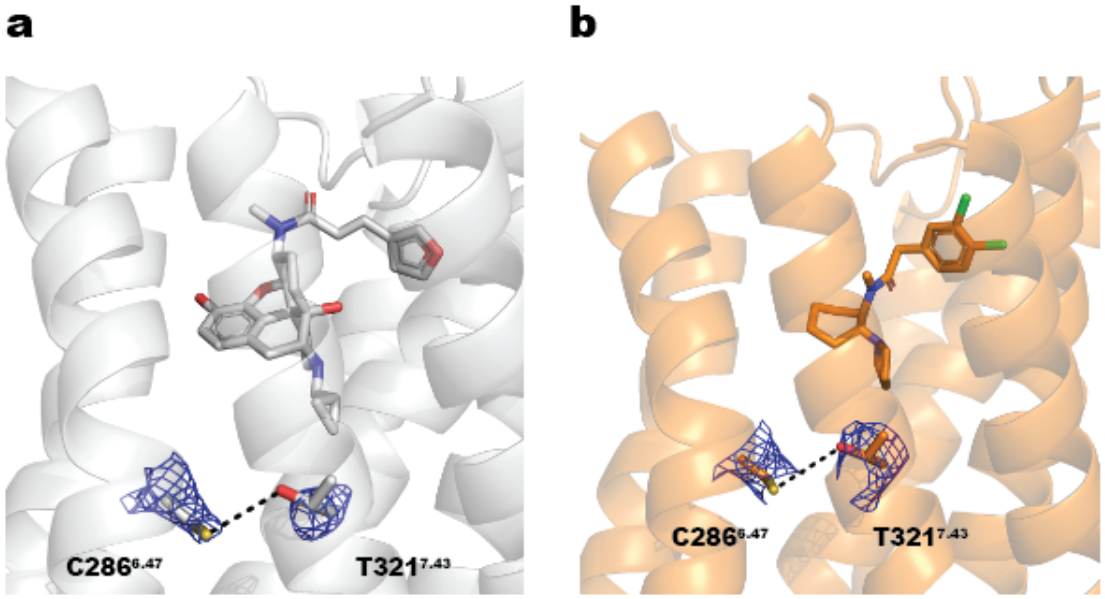


**Supplementary Figure 14. Interaction between the side chains of C286$^{6.47}$ and T321$^{7.43}$.** Interaction between C286$^{6.47}$ and T321$^{7.43}$ and conformational changes in TM6 during agonists and antagonist binding. The cryo-EM density is displayed as blue mesh.

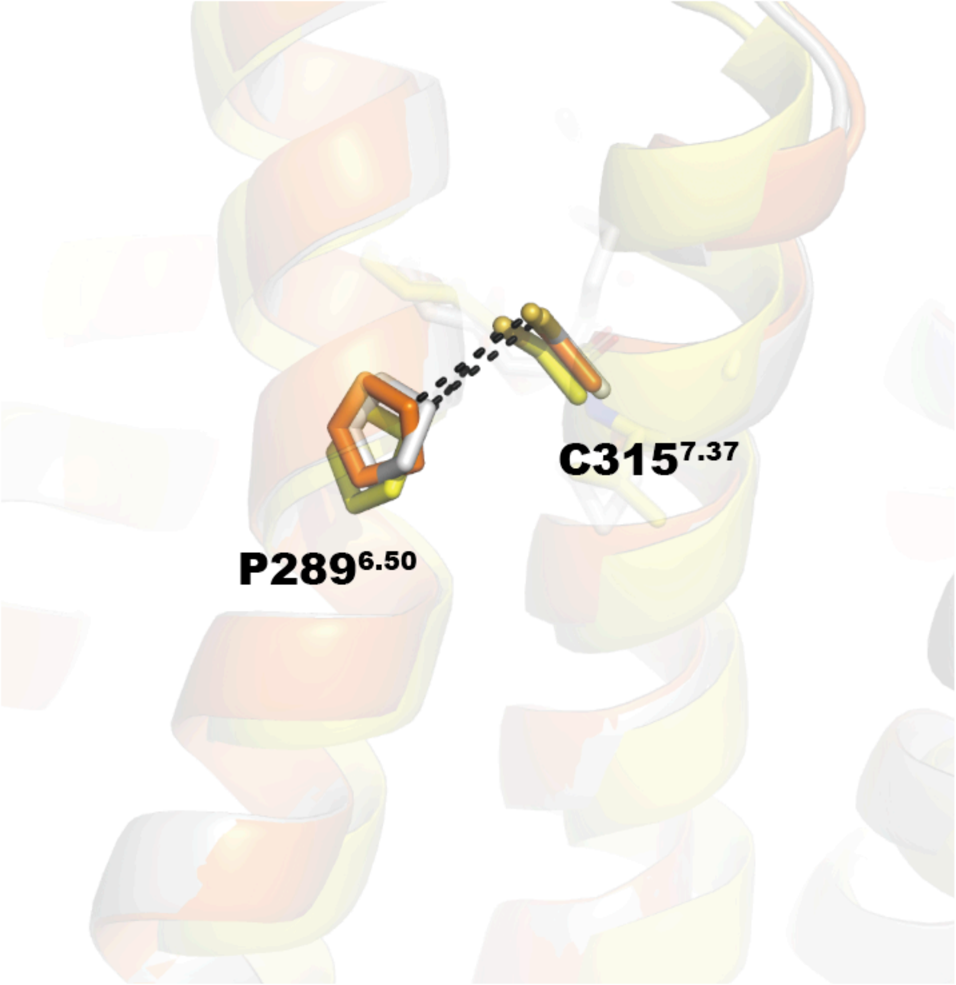


**Supplementary Figure 15. Interaction between the sidechains of P289$^{6.50}$ and C315$^{7.37}$.**

Nalfurafine-bound KORs are in gray, U-50,488H-bound KORs in orange and inverse agonist JDTic-bound KORs in yellow.

## KOR+Nalfurafine

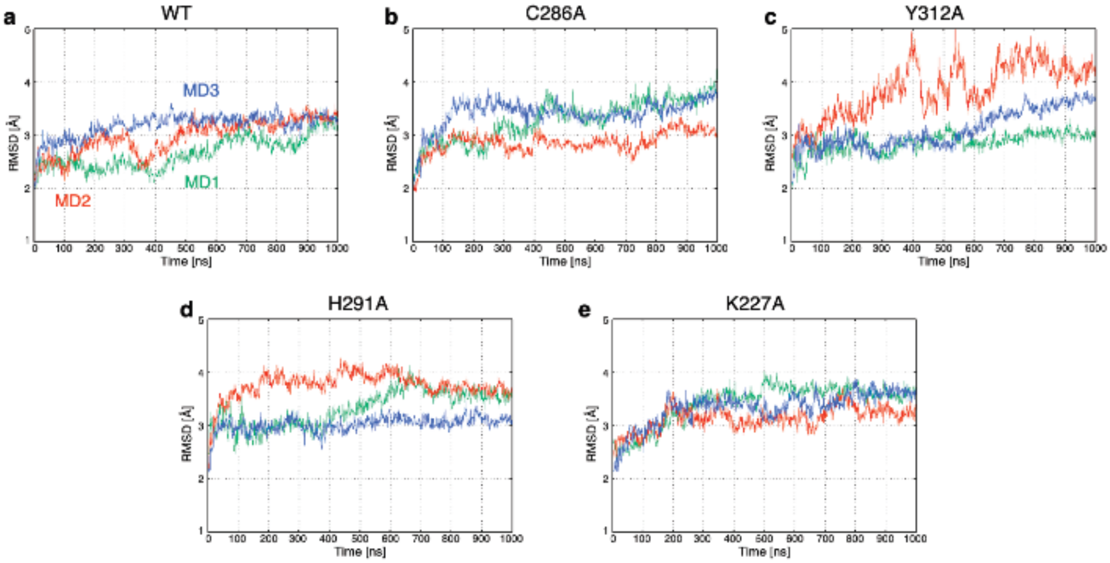


**Supplementary Figure 16. MD simulation of KOR–ligand complexes.**

RMSD of all heavy atoms of KOR and Nalfurafine, calculated using the initial conformation as a reference.

R(3.50)

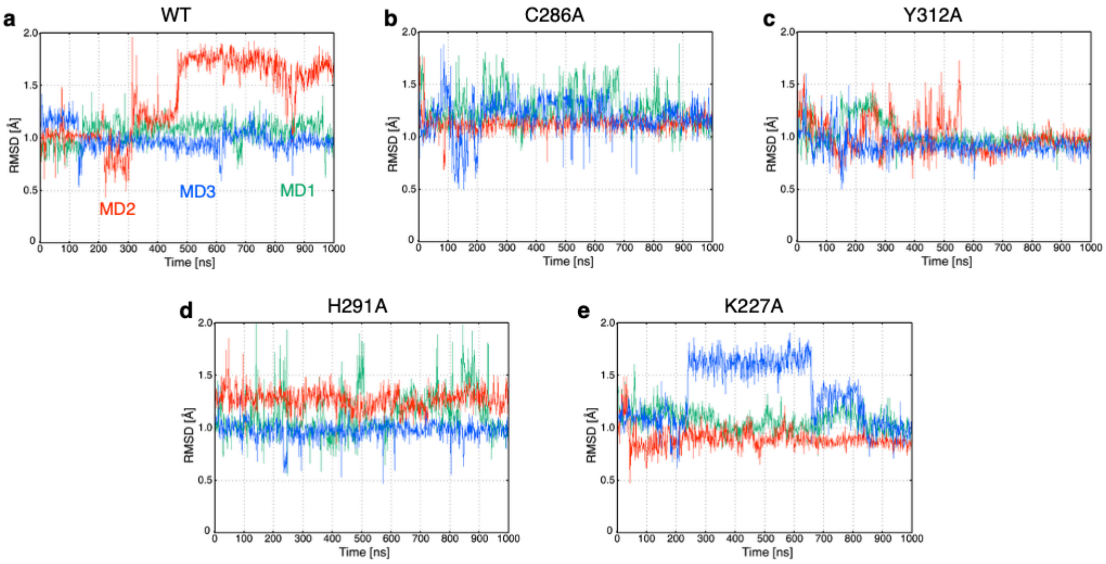


**Supplementary Figure 17. MD simulation of KOR–ligand complexes.**
RMSD of all heavy atoms of $R156^{3.50}$, calculated using the initial conformation as a reference.

Y(7.53)

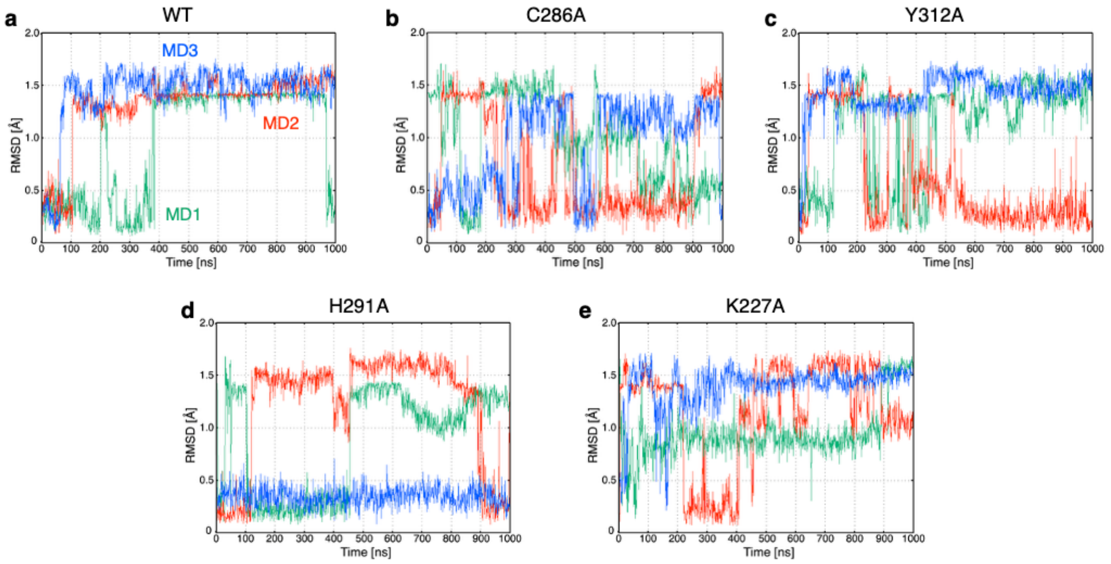


**Supplementary Figure 18. MD simulation of KOR–ligand complexes.**
RMSD of all heavy atoms of Y330$^{7.53}$, calculated using the initial conformation as a reference.

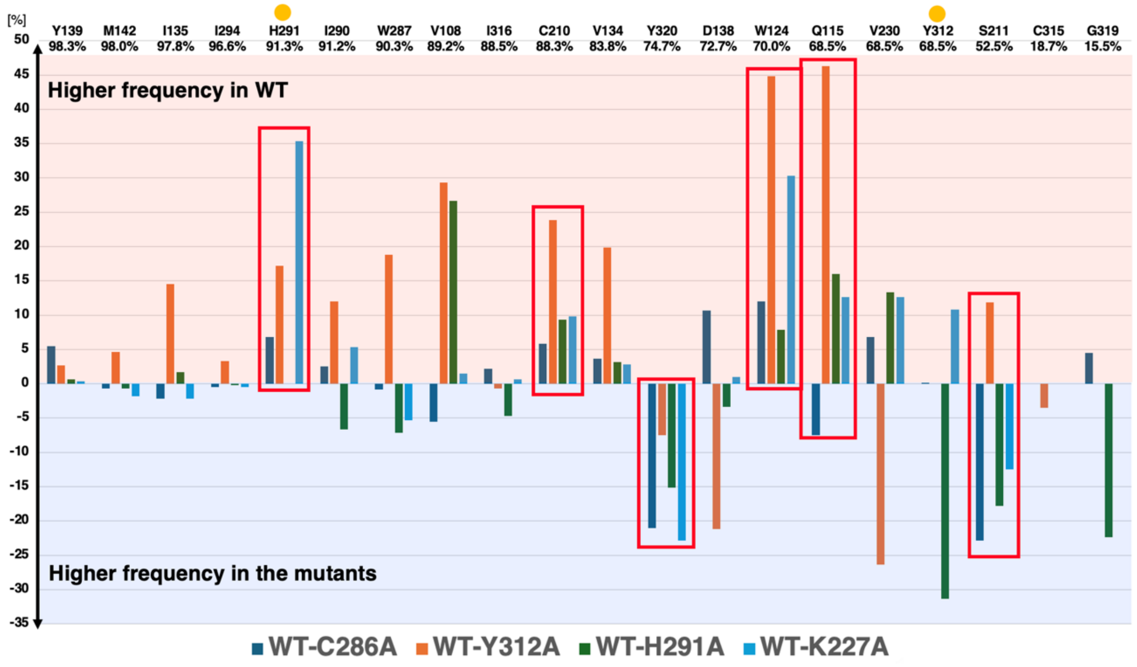


**Supplementary Figure 19. Differences in interaction frequencies between KOR and nalfurafine.**

Interaction frequency differences were calculated as the interaction frequency in the wild-type minus that in each of the four mutants. Numbers shown below each amino acid residue indicate the interaction frequency in the WT. Interaction fingerprint analysis was used to identify the amino acid residues within 6 Å of the ligand. Positive values indicate a reduction in interaction due to the mutations, while negative values indicate an increase.

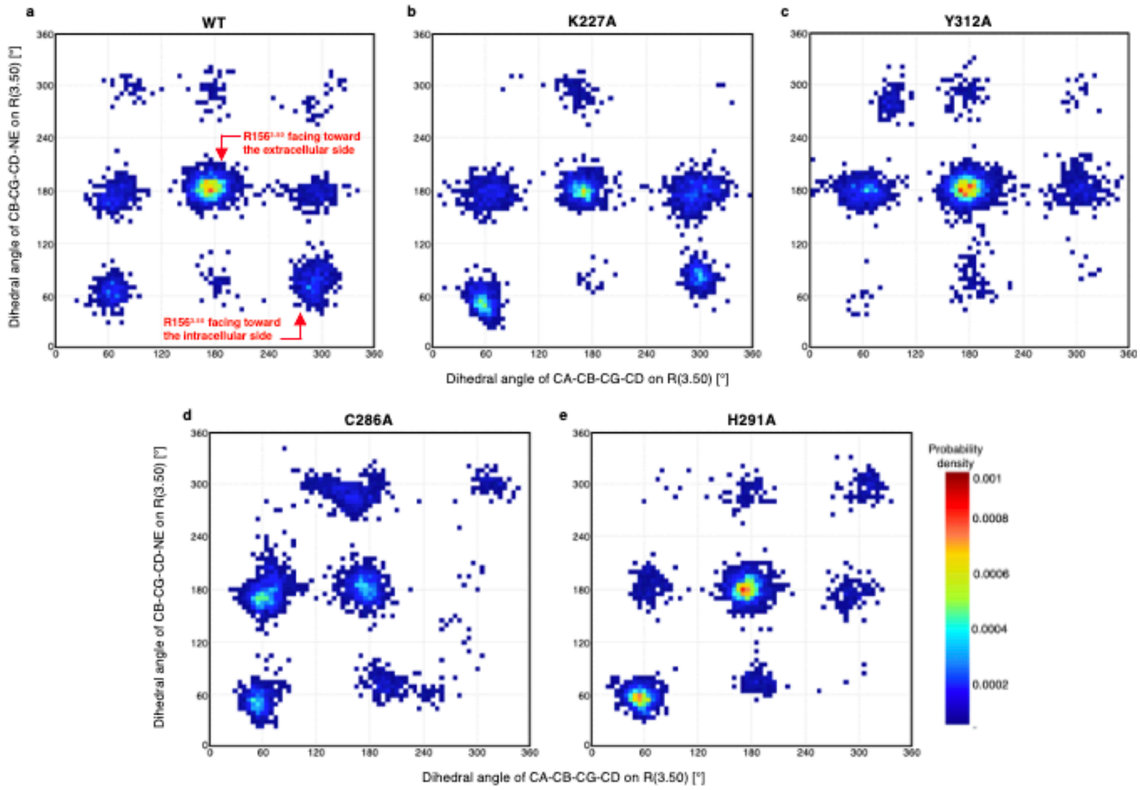


**Supplementary Figure 20. MD simulation of KOR–ligand complexes.**

Quantitative evaluation of R156$^{3.50}$ conformation using its dihedral angles in the MD simulations for the WT **(a),** K227$^{5.40}$A **(b)**, Y312$^{7.34}$A **(c)**, C286$^{6.47}$A **(d)**, and H291$^{6.52}$A **(e)**.

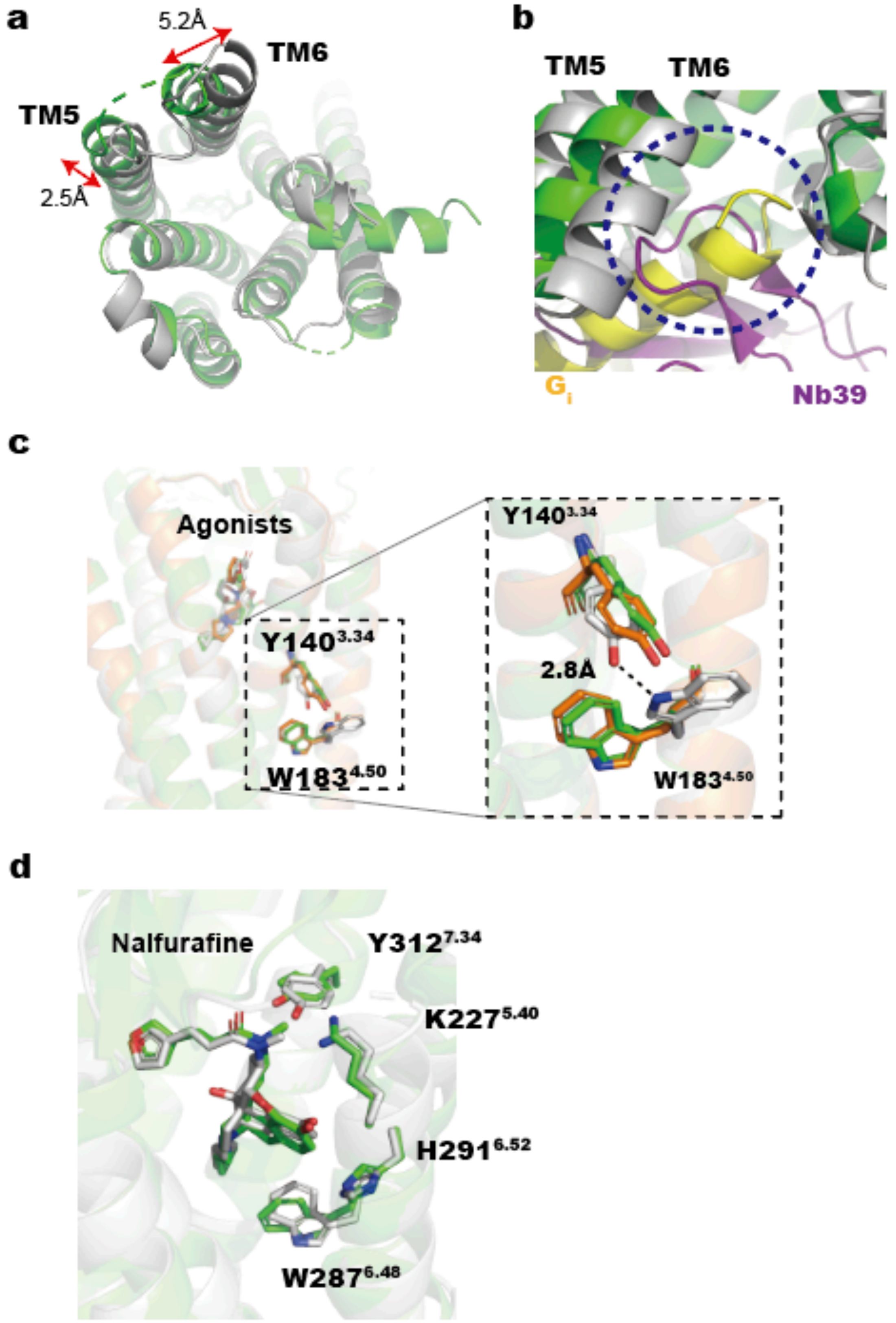


**Supplementary Figure 21. Structural comparison of KOR-Nb39 complex and KOR-G**

**protein complex in the nalfurafine-bound state.**

Superimposed view of the receptor region of the KOR-$G_i$ signaling complex (gray) and the KOR-Nb39 complex (green) in the nalfurafine-bound state from the intracellular side (**a**) and magnified view of the c-terminal helix of the G protein inserting into KOR and the binding site of Nb39 (**b**). Differences in the orientation of the side chains of $Y140^{3.34}$ and $W183^{4.50}$ are seen in the superposition diagram of the KOR-$G_i$ signaling complex bound with nalfurafine and U-50,488H (orange) and the KOR-Nb39 complex bound with nalfurafine **(c)**. Different orientations of the side chains of amino acids involved in β-arrestin-recruitment activity are found in this study **(d)**. In the nalfurafine-bound KOR-$G_i$ signaling complex, the space between interacting amino acids is indicated by a black dotted line.

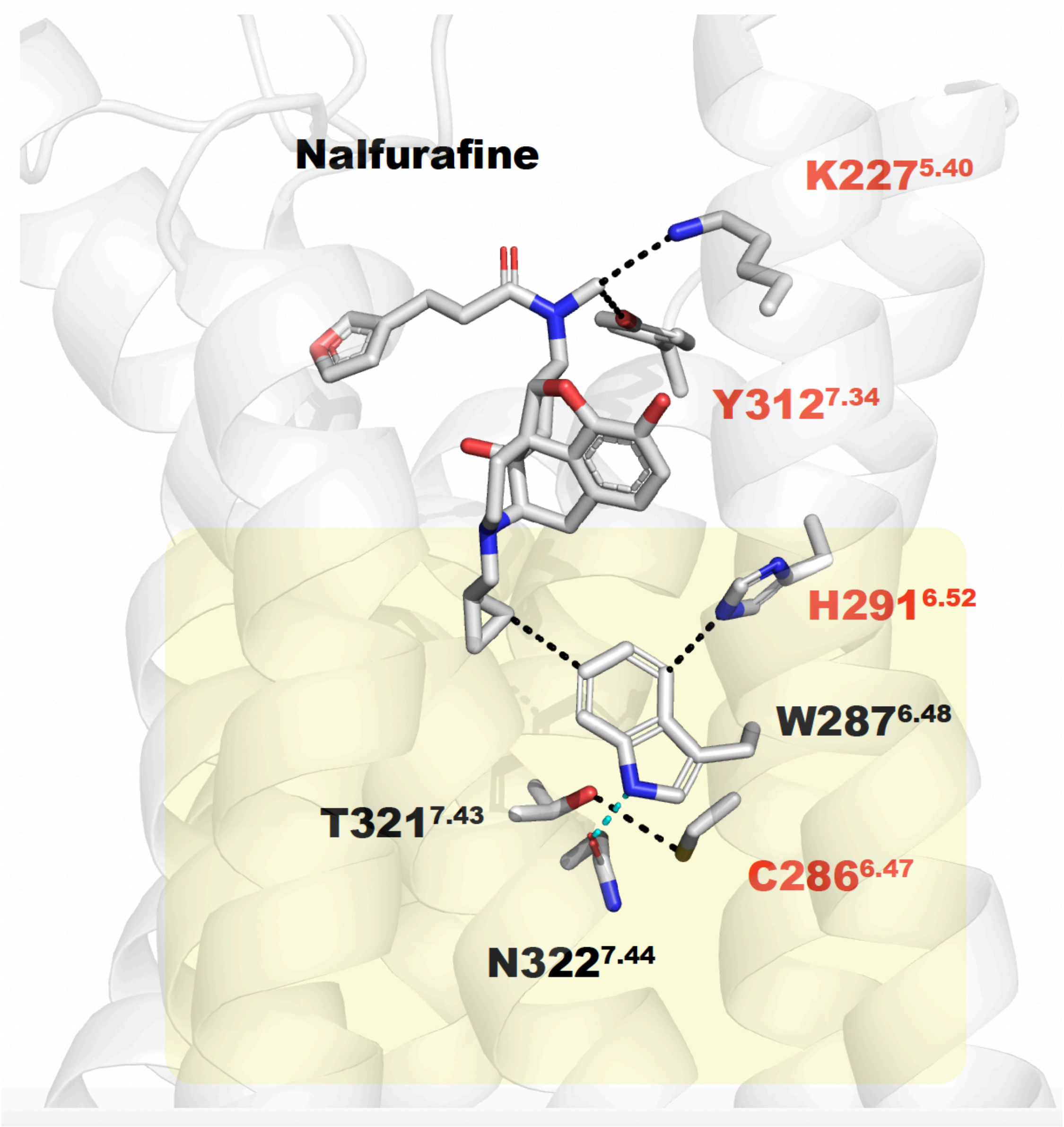


**Supplementary Figure 22. Amino acid residues within TM5, 6, and 7 that are involved in β-arrestin recruitment in KOR**

K227$^{5.40}$ and Y312$^{7.34}$ interact directly with the agonist, facilitating signal transducer-specific binding. On the contrary, alterations within the network of amino acid residues between TM6 and TM7 (highlighted in yellow) influence the selective binding of signaling factors. Amino acid residues crucial for β-arrestin recruitment, as identified in this study, are denoted by red letters.

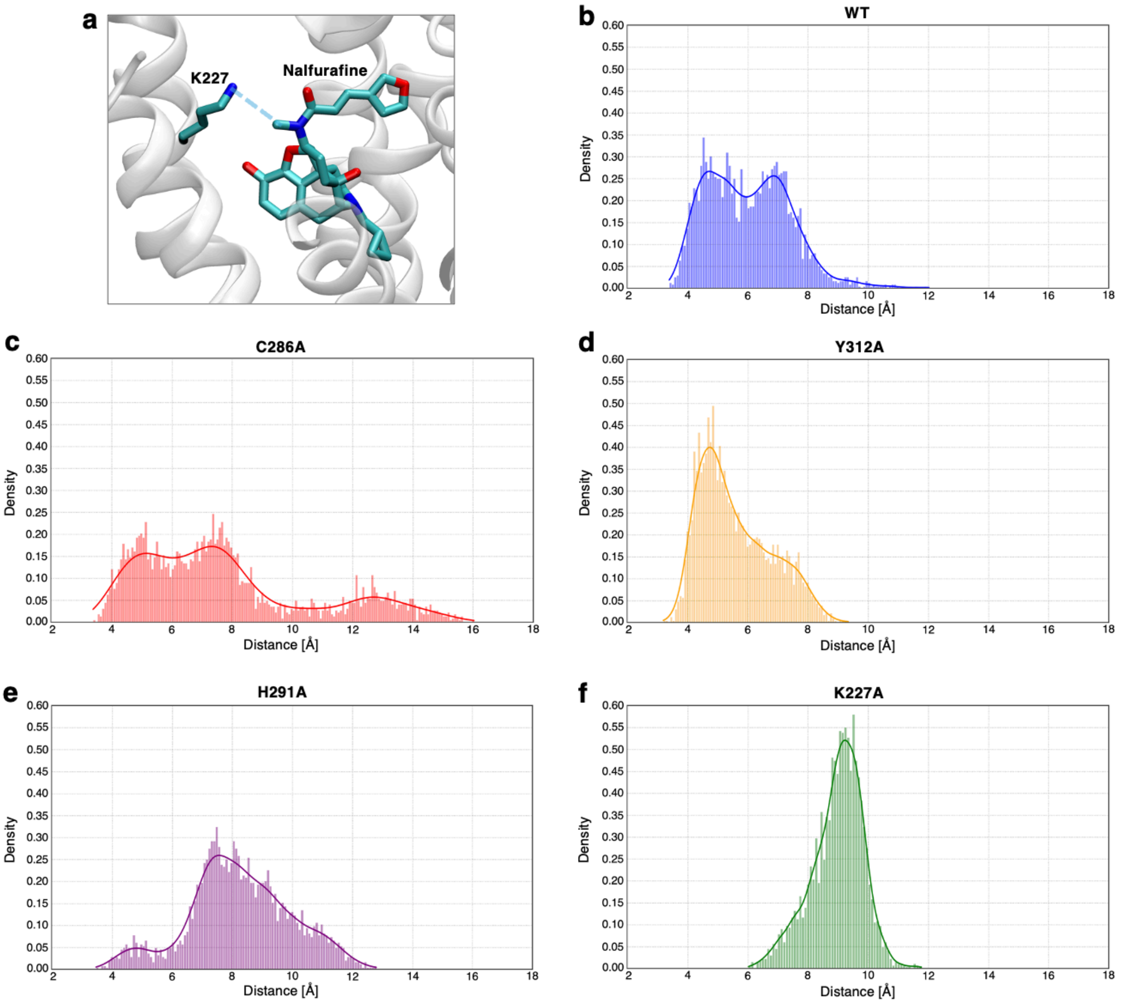


**Supplementary Figure 23. Interactions between K227$^{5.40}$ and nalfurafine in the MD simulations.**

The probability distributions of the interaction shown in panel **(a)** were calculated in the MD simulations for the WT **(b)**, K227$^{5.40}$A **(c)**, Y312$^{7.34}$A **(d)**, C286$^{6.47}$A **(e)**, and H291$^{6.52}$A **(f)**.

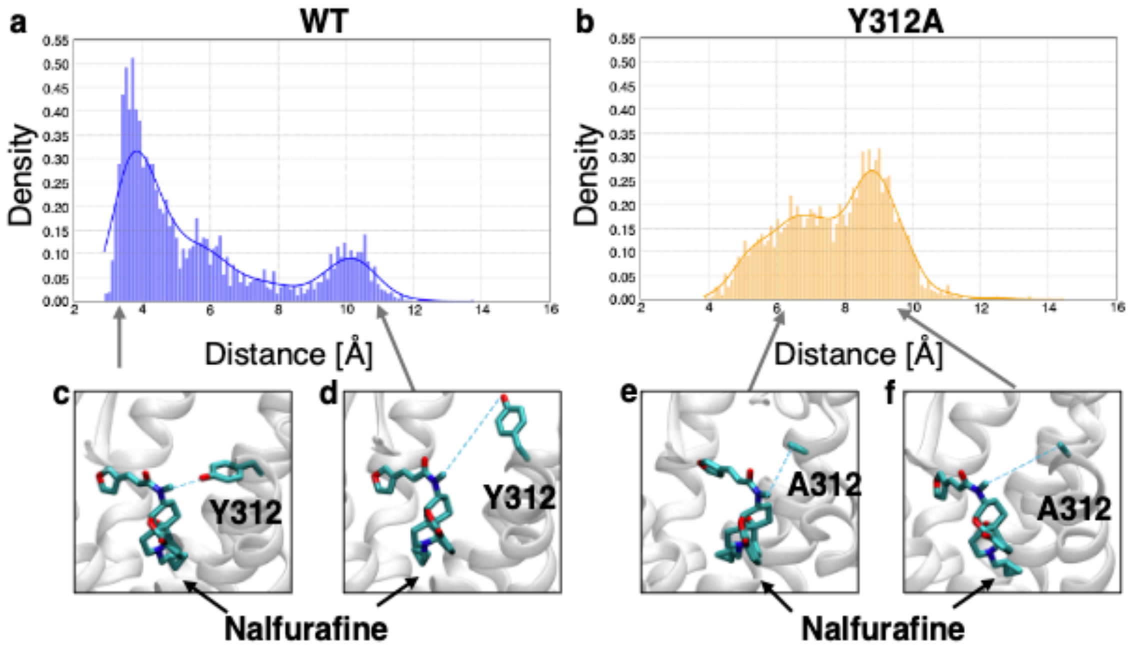


**Supplementary Figure 24. Probability distributions of the distances between the Y312$^{7.34}$ side chain and nalfurafine for the WT (a), and between the A312$^{7.34}$ side chain and nalfurafine for the Y312$^{7.34}$A mutant (b), as obtained from MD simulations.**

Snapshots illustrating specific distances are shown for the WT **(c, d)** and the Y312$^{7.34}$A mutant **(e, f)**

**Supplementary Table 1. Pharmacological parameters for the $G_i$-coupling activity and β-arrestin-recruiting activity.**

Pharmacological parameters for the $G_i$-coupling activity analyzed by the NanoBiT-G-protein dissociation assay and β-arrestin2-recruiting activity analyzed by the NanoBiT-β-arrestin recruitment assay. Data are presented as mean values ± SEM ($n$ = 3-4; dots). For the individual experiments performed in parallel, data were normalized to the wild-type (WT) KOR (1:1) and presented as $E_{max}$ and $\Delta pEC_{50}$. Statistical analyses were performed using the ordinary one-way ANOVA followed by Dunnett tests with the expression-matched (colored) WT response. *ns*, $p > 0.05$; $^{*}p < 0.05$; $^{**}p < 0.01$; $^{***}p < 0.001$. The exact P values, including those corresponding to Supplementary Table 1, are provided in the Source Data.

$G_i$ dissociation assay

| | Nalfurafine | | | | | | U-50,488H | | | | | |
|---|---|---|---|---|---|---|---|---|---|---|---|---|
| | *Emax* (%WT) | | | pEC50 | | | *Emax* (%WT) | | | pEC50 | | |
| | Mean | SEM | | Mean | SEM | | Mean | SEM | | Mean | SEM | |
| WT(1:1) | 100.0 | 0.0 | - | 10.22 | 0.21 | - | 100.0 | 0.0 | - | 8.69 | 0.08 | - |
| WT(1:2) | 100.0 | 1.0 | - | 10.11 | 0.11 | - | 100.4 | 0.7 | - | 8.63 | 0.05 | - |
| WT(1:4) | 98.3 | 0.8 | - | 9.96 | 0.12 | - | 97.6 | 1.6 | - | 8.42 | 0.02 | - |
| WT(1:8) | 90.5 | 2.1 | - | 9.86 | 0.08 | - | 93.2 | 0.8 | - | 8.14 | 0.02 | - |
| WT(1:16) | 77.8 | 1.0 | - | 9.83 | 0.07 | - | 82.4 | 0.1 | - | 7.92 | 0.06 | - |
| V108A | 102.8 | 1.1 | *ns* | 9.95 | 0.08 | *ns* | 97.6 | 1.4 | *ns* | 8.48 | 0.09 | *ns* |
| Y140F | 93.1 | 1.0 | * | 10.05 | 0.07 | *ns* | 88.6 | 1.3 | ** | 9.23 | 0.10 | *ns* |
| W183A | 101.1 | 2.5 | *ns* | 9.62 | 0.06 | *ns* | 97.6 | 2.0 | *ns* | 8.93 | 0.09 | *ns* |
| K227A | 101.7 | 0.6 | *ns* | 10.08 | 0.03 | *ns* | 101.9 | 0.4 | *ns* | 8.11 | 0.08 | *ns* |
| C229A | 101.4 | 0.7 | *ns* | 10.07 | 0.04 | *ns* | 101.6 | 1.5 | *ns* | 8.94 | 0.07 | *ns* |
| C286A | 99.0 | 1.1 | *ns* | 9.74 | 0.05 | *ns* | 96.8 | 0.4 | *ns* | 8.39 | 0.06 | *ns* |
| H291A | 93.6 | 0.3 | *ns* | 9.33 | 0.10 | ** | 93.3 | 1.9 | *ns* | 7.36 | 0.07 | *** |
| I294A | 92.6 | 1.5 | * | 9.32 | 0.36 | ** | 92.7 | 1.8 | * | 7.69 | 0.28 | ** |
| Y312A | 95.8 | 3.8 | *ns* | 8.90 | 0.09 | *** | 100.1 | 2.4 | *ns* | 6.73 | 0.22 | *** |
| Y312F | 103.0 | 1.8 | *ns* | 9.56 | 0.02 | * | 104.3 | 1.8 | *ns* | 7.46 | 0.18 | *** |
| C315A | 103.1 | 1.1 | *ns* | 9.99 | 0.18 | * | 102.9 | 1.3 | *ns* | 8.05 | 0.05 | ** |

β-arrestin 2 recruitment assay

| | Nalfurafine | | | | | | U-50,488H | | | | | |
|---|---|---|---|---|---|---|---|---|---|---|---|---|
| | *Emax* (%WT) | | | pEC50 | | | *Emax* (%WT) | | | pEC50 | | |
| | Mean | SEM | | Mean | SEM | | Mean | SEM | | Mean | SEM | |
| WT(1:1) | 100.0 | 0.0 | - | 8.73 | 0.05 | - | 100.0 | 0.0 | - | 7.51 | 0.12 | - |
| WT(1:2) | 101.7 | 4.2 | - | 8.69 | 0.06 | - | 103.4 | 4.5 | - | 7.53 | 0.14 | - |
| WT(1:4) | 104.2 | 6.8 | - | 8.73 | 0.03 | - | 114.1 | 4.7 | - | 7.50 | 0.09 | - |
| WT(1:8) | 100.0 | 10.2 | - | 8.80 | 0.01 | - | 111.8 | 13.0 | - | 7.51 | 0.13 | - |
| WT(1:16) | 105.4 | 13.4 | - | 8.86 | 0.01 | - | 123.5 | 16.3 | - | 7.51 | 0.13 | - |
| V108A | 103.7 | 4.3 | *ns* | 8.60 | 0.02 | *ns* | 124.4 | 6.7 | *ns* | 6.94 | 0.10 | *** |
| Y140F | 89.2 | 3.2 | *ns* | 8.63 | 0.06 | *ns* | 95.0 | 6.0 | *ns* | 7.80 | 0.08 | * |
| W183A | 80.9 | 3.1 | * | 8.34 | 0.07 | *** | 107.0 | 3.2 | *ns* | 7.48 | 0.17 | *ns* |
| K227A | 34.1 | 3.0 | * | 9.11 | 0.01 | * | 46.0 | 5.5 | * | 7.08 | 0.12 | * |
| C229A | 93.2 | 3.4 | *ns* | 8.78 | 0.04 | *ns* | 105.3 | 4.4 | *ns* | 7.57 | 0.14 | *ns* |
| C286A | 26.9 | 5.3 | ** | 8.94 | 0.08 | *ns* | 32.4 | 6.7 | * | 7.43 | 0.15 | *ns* |
| H291A | 51.8 | 4.1 | ** | 7.80 | 0.08 | ** | 39.2 | 4.1 | ** | 6.43 | 0.18 | ** |
| I294A | 91.5 | 4.9 | *ns* | 7.69 | 0.01 | *** | 87.3 | 1.8 | *ns* | 6.99 | 0.06 | *** |
| Y312A | 28.3 | 3.2 | *** | 8.33 | 0.13 | *ns* | 41.3 | 9.8 | ** | 5.79 | 0.05 | *** |
| Y312F | 38.7 | 11.3 | ** | 8.50 | 0.10 | *ns* | 66.1 | 21.2 | *ns* | 5.75 | 0.10 | *** |
| C315A | 89.7 | 5.5 | *ns* | 8.64 | 0.04 | *ns* | 104.3 | 8.0 | *ns* | 6.79 | 0.10 | *** |

## Supplementary Table 2 MD Simulation Overview.

| System | Replicas | Time length for each simulation [µs] | Box dimensions [Å] | Total number of atoms | Total number of water molecules | Salt concentration [mM] | Membrane lipids |
|---|---|---|---|---|---|---|---|
| WT | 3 | 1 | 119.9 × 119.9 × 128.2 | 141892 | 31386 | 150 | POPC (160:160) |
| $K227^{5.40}A$ | 3 | 1 | 119.9 × 119.9 × 128.0 | 141624 | 31300 | 150 | POPC (160:160) |
| $Y312^{7.53}A$ | 3 | 1 | 119.9 × 119.9 × 128.1 | 141549 | 31275 | 150 | POPC (160:160) |
| $C286^{6.47}A$ | 3 | 1 | 119.9 × 119.9 × 128.0 | 141588 | 31284 | 150 | POPC (160:160) |
| $H291^{6.52}A$ | 3 | 1 | 119.9 × 119.9 × 128.0 | 141604 | 31292 | 150 | POPC (160:160) |

**Supplementary Table 3 Cryo-EM data collection, refinement, and validation statistics**

| | U-50,488H bound KOR-$G_i$ signaling complex (EMD-65622) (PDB: 9W49) | Nalfurafine bound KOR-$G_i$ signaling complex (EMD-64947) (PDB: 9V6O) |
|---|---|---|
| **Data collection and processing** | | |
| Magnification | 105,000 | 105,000 |
| Voltage (keV) | 300 | 300 |
| Electron exposure ($e^-/Å^2$) | 60 | 60 |
| Defocus range (μm) | -0.7 to -1.5 | -0.7 to -1.5 |
| Pixel size (Å) | 0.675 | 0.675 |
| Symmetry imposed | C1 | C1 |
| Initial particle images (no.) | 4,148,764 | 7,066,947 |
| Final particle images (no.) | 1,225,096 | 858,423 |
| Map resolution (Å) | 2.90 | 2.76 |
| FSC threshold | 0.143 | 0.143 |
| Map resolution range (Å) | 2.47-14.99 | 2.36-6.67 |
| **Refinement** | | |
| Initial model used (PDB code) | 7YIT(KOR), 6CMO ($Gα_i$, scFv16), 6DDE (Gβ, Gγ) | 7YIT(KOR), 6CMO ($Gα_i$, scFv16), 6DDE (Gβ, Gγ) |
| Model resolution (Å) | 2.90 | 2.76 |
| FSC threshold | 0.5 | 0.5 |
| Map sharpening *B* factor ($Å^2$) | 128.4 | 110.4 |
| Model composition | | |
| Non-hydrogen atoms | 8,284 | 8,358 |
| Protein residues | 1,103 | 1,107 |
| Ligands | 1 | 1 |
| *B* factors ($Å^2$) | | |
| Protein | 71.10 | 58.51 |
| Ligand | 112.45 | 80.37 |
| R.m.s. deviations | | |
| Bond lengths (Å) | 0.003 | 0.003 |
| Bond angles (°) | 0.531 | 0.492 |

| | | |
|---|---|---|
| Validation | | |
| MolProbity score | 2.37 | 1.93 |
| Clashscore | 9.63 | 6.11 |
| Poor rotamers (%) | 8.47 | 4.05 |
| Ramachandran plot | | |
| Favored (%) | 97.13 | 97.33 |
| Allowed (%) | 2.87 | 2.67 |
| Disallowed (%) | 0 | 0 |
