# Supplementary material for: Structural and dynamic insights into the biased signaling mechanism of the human kappa opioid receptor
Source: Nat Commun. 2025 Oct 28;16:9392. doi: 10.1038/s41467-025-64882-1 (PMC12569041; doi:10.1038/s41467-025-64882-1)
Supplement: Supplementary file 2 — Description of Additional Supplementary Files [file 41467_2025_64882_MOESM2_ESM.pdf]

Description of Additional Supplementary Files

File Name: Supplementary Data 1
Description: Pharmacological parameters for the Gi-coupling activity and β-arrestin-recruiting activity.
