# Supplementary material for: Structural and dynamic insights into the biased signaling mechanism of the human kappa opioid receptor
Source: Nat Commun. 2025 Oct 28;16:9392. doi: 10.1038/s41467-025-64882-1 (PMC12569041; doi:10.1038/s41467-025-64882-1)
Supplement: Supplementary file 4 — Reporting Summary [file 41467_2025_64882_MOESM4_ESM.pdf]

nature portfolio

Corresponding author(s): Takuya Kobayashi and Ryoji Suno

Last updated by author(s): Aug 15, 2025

Reporting Summary

Nature Portfolio wishes to improve the reproducibility of the work that we publish. This form provides structure for consistency and transparency in reporting. For further information on Nature Portfolio policies, see our Editorial Policies and the Editorial Policy Checklist.

Statistics

For all statistical analyses, confirm that the following items are present in the figure legend, table legend, main text, or Methods section.

n/a	Confirmed	
☐	☒	The exact sample size (*n*) for each experimental group/condition, given as a discrete number and unit of measurement
☐	☒	A statement on whether measurements were taken from distinct samples or whether the same sample was measured repeatedly
☐	☒	The statistical test(s) used AND whether they are one- or two-sided<br>*Only common tests should be described solely by name; describe more complex techniques in the Methods section.*
☒	☐	A description of all covariates tested
☒	☐	A description of any assumptions or corrections, such as tests of normality and adjustment for multiple comparisons
☐	☒	A full description of the statistical parameters including central tendency (e.g. means) or other basic estimates (e.g. regression coefficient) AND variation (e.g. standard deviation) or associated estimates of uncertainty (e.g. confidence intervals)
☐	☒	For null hypothesis testing, the test statistic (e.g. *F*, *t*, *r*) with confidence intervals, effect sizes, degrees of freedom and *P* value noted<br>*Give P values as exact values whenever suitable.*
☒	☐	For Bayesian analysis, information on the choice of priors and Markov chain Monte Carlo settings
☒	☐	For hierarchical and complex designs, identification of the appropriate level for tests and full reporting of outcomes
☒	☐	Estimates of effect sizes (e.g. Cohen's *d*, Pearson's *r*), indicating how they were calculated

Our web collection on statistics for biologists contains articles on many of the points above.

Software and code

Policy information about availability of computer code

Data collection	cryoEM data were collected using serialEM 4.0.
Data analysis	Graphpad Prism ver. 8 was used for all statistical analysis for the in vivo assay experiments. cryoEM data processing was done in cryoSPARC. Model building was done using Phenix1.20-4459 and Coot0.9.8.7. Figures were made using UCSF Chimera1.8 and PyMOL2.5.0. CHARMM-GUI builder was used to model and embed the receptor in a lipid bilayer.<br>All non-biased simulations were performed using Amber20.<br>The software VMD1.9.4 and AmberTools20 were used to visualize and analyze MD simulations.

For manuscripts utilizing custom algorithms or software that are central to the research but not yet described in published literature, software must be made available to editors and reviewers. We strongly encourage code deposition in a community repository (e.g. GitHub). See the Nature Portfolio guidelines for submitting code & software for further information.

Data

Policy information about availability of data

All manuscripts must include a data availability statement. This statement should provide the following information, where applicable:

- Accession codes, unique identifiers, or web links for publicly available datasets
- A description of any restrictions on data availability
- For clinical datasets or third party data, please ensure that the statement adheres to our policy

All data generated, analyzed in this study, methods used are included in the Supplementary Information. The cryo-EM maps and corresponding coordinates have

been deposited in the Electron Microscopy Data Bank (EMDB) and the Protein Data Bank (PDB), respectively, under the following accession codes: EMD-64947 and 9V6O(Nalfurafine bound KOR-Gi signaling complex), EMD-65622 and 9W49(U-50,488H bound KOR-Gi signaling complex).
The authors declare that all data supporting the findings of this study are available in the article and the Supplementary information file, including data of signaling assays of kappa opioid receptor. All raw data for all figures is available in the source data files. The crystal structure of the kappa-opioid receptor was obtained from RCSB PDB using entry ID 7YIT. All compounds can be made available on reasonable requests from the authors.

Research involving human participants, their data, or biological material

Policy information about studies with human participants or human data. See also policy information about sex, gender (identity/presentation), and sexual orientation and race, ethnicity and racism.

Reporting on sex and gender	N/A.
Reporting on race, ethnicity, or other socially relevant groupings	N/A.
Population characteristics	N/A.
Recruitment	N/A.
Ethics oversight	N/A.

Note that full information on the approval of the study protocol must also be provided in the manuscript.

Field-specific reporting

Please select the one below that is the best fit for your research. If you are not sure, read the appropriate sections before making your selection.

☒ Life sciences ☐ Behavioural & social sciences ☐ Ecological, evolutionary & environmental sciences

For a reference copy of the document with all sections, see nature.com/documents/nr-reporting-summary-flat.pdf

Life sciences study design

All studies must disclose on these points even when the disclosure is negative.

Sample size	No statiscal method was used to determine the sample size. We performed three in dependent experiments.<br>d three
Data exclusions	No data were excluded for this study.
Replication	In vitro experiments were independently performed at least three times for all of the other experiments.
Randomization	No randomization was attempted or needed. This was not a clinical trial or animal study that is dependent on randomization.
Blinding	No blinding was attempted or needed.

Reporting for specific materials, systems and methods

We require information from authors about some types of materials, experimental systems and methods used in many studies. Here, indicate whether each material, system or method listed is relevant to your study. If you are not sure if a list item applies to your research, read the appropriate section before selecting a response.

Materials & experimental systems

n/a	Involved in the study	
☐	☒	Antibodies
☐	☒	Eukaryotic cell lines
☒	☐	Palaeontology and archaeology
☒	☐	Animals and other organisms
☒	☐	Clinical data
☒	☐	Dual use research of concern
☒	☐	Plants

Methods

n/a	Involved in the study	
☒	☐	ChIP-seq
☐	☒	Flow cytometry
☒	☐	MRI-based neuroimaging

Antibodies

Antibodies used	anti-FLAG epitope (DYKDDDDK) tag monoclonal antibody (Clone 1E6, FujiFilm Wako Pure Chemicals)

Validation	Anti-DYKDDDDKn gives a low signal (less than 1:100) in mock-transfected HEK 293 cells. The antibody was validated by the manufacture (https://labchem-wako.fujifilm.com/us/category/01430.html)

Eukaryotic cell lines

Policy information about cell lines and Sex and Gender in Research

Cell line source(s)	Sf9 insect cells were purchased from Expression Systems. HEK293
Authentication	Cells have not been authenticated after purchase.
Mycoplasma contamination	Cells have not been tested for mycoplasma.
Commonly misidentified lines (See ICLAC register)	No commonly misidentified cell lines were used in the study.

Plants

Seed stocks	N/A.
Novel plant genotypes	N/A.
Authentication	N/A.

Flow Cytometry

Plots

Confirm that:

- [x] The axis labels state the marker and fluorochrome used (e.g. CD4-FITC).
- [x] The axis scales are clearly visible. Include numbers along axes only for bottom left plot of group (a 'group' is an analysis of identical markers).
- [x] All plots are contour plots with outliers or pseudocolor plots.
- [x] A numerical value for number of cells or percentage (with statistics) is provided.

Methodology

Sample preparation	HEK293 cells were transfected by combining 2.5 μL of the polyethylenimine solution (1 mg mL-1) and 200 ng of a plasmid encoding FLAG epitope-tagged GPCR (volumes denote per well in 6-well plate).
Instrument	EC800 flow cytometer (Sony)
Software	FlowJo software (FlowJo)
Cell population abundance	N/A (since we analyzed a single cell type and obtained data from all observed cells.)
Gating strategy	N/A (since we used all of the recorded fluorescent signals and alculate a simple mean value, description of gating strategy or the plot representation was not performed).

- [ ] Tick this box to confirm that a figure exemplifying the gating strategy is provided in the Supplementary Information.
